# The Impairment of Social and Environmental Relationships in Patients With Heart Failure Correlated With Therapeutic Class

**DOI:** 10.7759/cureus.62775

**Published:** 2024-06-20

**Authors:** Diana Carina Iovanovici, Carmen Delia Nistor Cseppento, Delia Mirela Tit, Anamaria Lavinia Purza, Sebastian Tirla, Cristina Aur, Simona Gabriela Bungau

**Affiliations:** 1 Doctoral School of Biomedical Sciences, University of Oradea, Oradea, ROU; 2 Department of Psycho-Neuroscience and Rehabilitation, University of Oradea, Oradea, ROU; 3 Department of Pharmacy, University of Oradea, Oradea, ROU; 4 Department of Surgical Disciplines, University of Oradea, Oradea, ROU

**Keywords:** comorbidity, therapeutic class, environmental relationships, social relationships, heart failure

## Abstract

Background and objectives

Heart failure (HF) significantly influences the quality of life, both physically and emotionally, as well as social and environmental relationships. One major objective of HF treatment is to maintain or improve the quality of life. The aims of the study were to assess the impact of HF on social relationships and the relationship with the environment, according to therapeutic class and the presence of comorbidities, and to identify predictive factors for the impairment of these dimensions of the quality of life.

Materials and methods

This study was based on a cross-sectional survey; 252 patients with HF who have referred themselves to the medical rehabilitation department of the "Avram Iancu" Clinical Hospital, Oradea, between February 2023 and February 2024 were included. The patients were divided into two groups (Group HF-S/V, patients undergoing treatment with sacubitril/valsartan; Group HF-CT, patients receiving conventional therapy). All patients were asked to complete two assessment tools: the Charlson Comorbidity Index (CCI) questionnaire and the World Health Organization Quality of Life Brief Version (WHOQOL-BREF) questionnaire.

Results

The mean values obtained per the domain of social relationships were significantly better for Group HF-CT (65.762 ± 12.519 versus 61.266 ± 12.428, p = 0.024). The mean values obtained on the domain of social relations and in relation to the environment were significantly better for Group HF-CT (65.762 ± 12.519 versus 61.266 ± 12.428, p = 0.024; 61.333 ± 13.461 versus 51.719 ± 16.769, p < 0.001). Both dimensions of the quality of life correlate with age and CCI (F = 7.793, p < 0.001, for social relationships; F = 16.821, p < 0.001, for relationship with the environment).

Conclusions

Social relationships and the relationship with the environment are affected in HF patients and correlate with age and comorbidity index, regardless of the type of therapy.

## Introduction

Heart failure (HF), characterized by heart pump failure, is associated with increased morbidity and mortality [1]. Data show that in Romania, more than 4% of the population over 35 years old suffer from HF, with an annual mortality rate of about 60% and a 10-year survival rate of 10% [2]. HF is defined by characteristic symptoms caused by a "structural or functional abnormality," elevated natriuretic peptide levels, and impaired left ventricular ejection fraction (LVEF) [3]. Based on LVEF values, we have the following classification: LVEF ≥ 50%, HF with preserved ejection fraction (HFpEF); ejection fraction (EF) = 41%-49%, HF with mildly reduced EF (HFmrEF); and EF ≤ 40%, HF with reduced EF (HFrEF) [4].

The risk factors well recognized as being linked to the development of HF through a number of mechanisms are hypertension, type 2 diabetes, dyslipidemia, obesity, sedentary lifestyle, and smoking [5]. Psychosocial stress and environmental health impact cardiovascular risk factors and thus the progression of cardiovascular pathology [6].

Drugs used to treat HF are angiotensin-converting enzyme inhibitors (ACEIs), angiotensin II receptor antagonists (ARBs), mineralocorticoid receptor antagonists (MRAs), and isosorbide/hydralazine. In the 1980s, diuretics, digoxin, and inotropic vasodilators were recommended; milrinone, flosequinan, and beta-blockers appeared in the following years [7]. Depending on the symptoms and underlying cause of HF, different medications are prescribed. Only a few drugs are approved for the relief of symptoms of HF. The combination therapy sacubitril/valsartan (S/V) is one of the drugs considered for the relief of HF symptoms [7]. One major objective of HF treatment is to maintain or improve the quality of life, as many patients place a higher value on the quality of life than on longevity. The best indicators of low quality of life include depression, medical comorbidities, and HF symptoms and functional severity [8].

Comorbidities, defined as "the presence of more than one disorder in a person over a defined period" [9], significantly influence patients' quality of life; non-cardiac comorbidities influence hospital admission rates. The most common non-cardiac comorbidities are chronic kidney disease, diabetes mellitus, peripheral vascular disease, and dementia [9].

Through its severity and frequent association with other comorbidities, the presence of HF significantly influences the quality of life, in both the physical and emotional domains, as well as social and environmental relationships.

The aims of the study were to assess the impact of HF on social relationships and the relationship with the environment, according to different treatment strategies (conventional therapies {CT} versus S/V) and the presence of comorbidities, and to identify predictive factors for the impairment of these dimensions of the quality of life. This is the first study in Romania, to our knowledge, to assess these aspects in patients with HF.

## Materials and methods

Database

A cross-sectional survey and descriptive correlational design were used for this study. Data were obtained from a group of 252 HF patients, regardless of LVEF values, who have referred themselves to the medical rehabilitation department of the "Avram Iancu" Clinical Hospital, Oradea, between February 2023 and February 2024. All these patients had a definite diagnosis of HF. The selection of subjects led to the formation of two groups: Group HF-S/V, patients undergoing treatment with sacubitril/valsartan, associated or not with diuretics/beta-blockers/sodium-glucose cotransporter-2 (SGLT2) inhibitors, according to clinical particularities, and Group HF-CT, patients receiving conventional therapy, which included diuretics, converting enzyme inhibitors, beta-blockers, ARBs, and MRAs.

Inclusion/exclusion criteria

**Figure 1:**
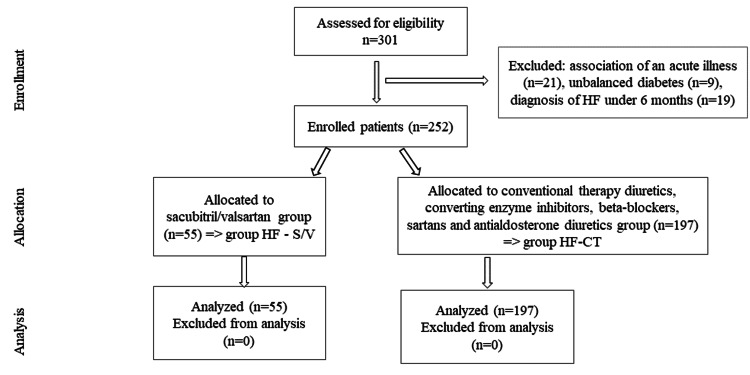
CONSORT flow diagram of the study. Group HF-S/V, patients undergoing treatment with sacubitril/valsartan; Group HF-CT, patients receiving conventional therapy CONSORT, Consolidated Standards of Reporting Trials; HF, heart failure

Patients with chronic HF, left HF, right HF, and total HF, with New York Heart Association (NYHA) stages I, II, and III and a definite diagnosis of more than six months, regardless of whether HF was the primary or secondary diagnosis were included. Patients under 18 years of age with decompensated forms of HF, patients with HF stage IV (where there is a clear deterioration in the quality of life in all domains) or decompensated forms of associated diseases, and patients with incomplete or inaccessible Charlson Comorbidity Index (CCI) variables were excluded. The comorbidities assessed were cardiovascular disease including acute myocardial infarction, kidney disease, diabetes mellitus, stroke, chronic lung disease, peptic ulcer, inflammatory rheumatism, cancer, leukemia, lymphoma, AIDS, and liver diseases as underlying diseases. Of the 301 patients recruited, 252 patients remained in the study. The allocation by study group is summarized in Figure 1.

Sample size

The sample size was calculated based on the total number of patients who were admitted during the study period, with a diagnosis of HF, according to the inclusion criteria. Several variables (p, the probability of the occurrence of the phenomenon, 0p1; q, counter-probability, q = 1 - p; t, probability factor, x - error limit; and N, community volume) were considered by calculating the minimum sample size. The formula used to estimate the minimum sample is n = t2 pq / (x2 + t2 pq / N) [10]. The value of n is maximum if the product of pq is maximum (p = q = 0.5). The 95% probability corresponds to a value of t = 1.96. A limiting error of 0.1 has been set. If N is large, above 10000 (in our case, N = 252), the ratio t2 pq / N is neglected. The result obtained by applying the above formula is N = 96.

Study tools

All patients were asked to complete two assessment tools: the Charlson Comorbidity Index (CCI) questionnaire and the World Health Organization Quality of Life Brief Version (WHOQOL-BREF) (license ID: 202300206).

The CCI is a clinimetric index that is calculated according to the number and severity of comorbidities [11]. The comorbidities that are scored are myocardial infarction, congestive HF, peripheral vascular disease, cerebrovascular disease, dementia, chronic lung disease, rheumatological diseases (systemic lupus erythematosus, polymyositis, rheumatoid arthritis, and polymyalgia rheumatica), ulcer disease, liver disease, and diabetes mellitus. In total, there are 17 items. Each item receives a score between 0 and 6, depending on the stage of the disease and its severity (for a localized tumor, two points are added and, for metastases, six points). The maximum value is 37; the score is adjusted according to age. After scoring each comorbidity, a 10-year survival rate can be calculated. We used the online calculator to determine the CCI score [11]. In order to quantify the data more efficiently [12], three grades of comorbidity severity were considered: mild, with CCI scores of 1-2; moderate, with CCI scores of 3-4; and severe, with CCI scores of ≥5.

**Figure 2:**
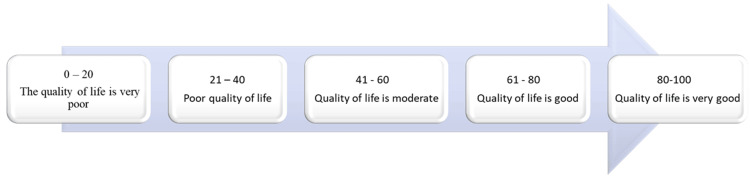
Interpretation of the results of the World Health Organization Quality of Life Brief Version (WHOQOL-BREF) questionnaire.

The WHOQOL-BREF is a self-report questionnaire that assesses several dimensions of an individual's life in four domains: physical, psychological, social relationships, and relationship with the environment [13]. This study focuses on assessing the last two domains. The WHOQOL-BREF consists of 26 items; the first two questions refer to "individual's general perception of quality of life" (Q1) and the second to "individual's general perception of their health" (Q2). The evaluation of social relations includes three questions; eight questions are used to evaluate the relationship with the environment. The interpretation of the results is done using the Likert scale [14]. The score is between 0 (lowest health status) and 100 (best health status) (Figure 2). Values can be calculated for each domain separately. The figures obtained per domain are converted on a scale from 0 to 100; thus, the number obtained is multiplied by 6.25 [15].

Ethical approval

The Research Ethics Commission of the Faculty of Medicine and Pharmacy of the University of Oradea issued approval CEFMF/02/19.05.2022. The research was conducted according to the World Medical Association Declaration of Helsinki guidelines. Participation in the study was voluntary. Written informed consent was obtained from all participants.

Statistical analysis

Data processing was performed using JASP version 0.18.1.0. Mean values of parameters, frequency ranges, standard deviations, and statistical significance tests using Student's method (t-test) were used to compare means, and the level of statistical significance was 0.05. Levene's test was used to assess the homogeneity of dispersion. If the homogeneity of dispersion was not respected between the two groups and the variances were significantly different, the Mann-Whitney U test was used to compare the two groups. To compare other distributions between groups, we used the chi-square method. A multiple linear regression model was fitted to identify factors influencing the quality of life score.

## Results

Baseline characteristics

The main characteristics of the patients included in the study are shown in Table 1. The two groups are relatively homogeneous in terms of demographic characteristics, with a higher frequency of male and urban patients in both groups. The clinical parameters assessed differed within the two groups, especially for HF, high blood pressure (HBP), dyslipidemia, dilated cardiomyopathy, chronic kidney disease, and lung disease.

**Table 1:** Baseline characteristics of the groups. Group HF-S/V, patients undergoing treatment with sacubitril/valsartan; Group HF-CT, patients receiving conventional therapy *T-test **Chi-square SD, standard deviation; HF, heart failure; HBP, high blood pressure; LVEF, left ventricle ejection fraction; NYHA, New York Heart Association

Parameter	Group HF-CT	Group HF-S/V	P-value
Age, Mean, SD (N)	63.467 ± 11.509 (197)	66.327 ± 11.246 (55)	0.103*
Male, N, %	100 (50.76)	34 (61.81)	0.099**
Rural area, N (%)	57 (28.93)	25 (45.45)	0.021**
Weight, N (%)	197 (100)	55 (100)	0.338**
Normal weight, N (%)	6 (10.90)	36 (18.27)	<0.001**
Overweight, N (%)	62 (31.47)	20 (36.36)	<0.001**
Grade I obesity, N (%)	56 (28.42)	21 (38.18)	0.0001
Grade II obesity, N (%)	30 (15.22)	8 (14.54)	0.0004
Grade III obesity, N (%)	11 (5.58)	-	-
Underweight, N (%)	2 (1.01)	-	-
HF, N (%)	197 (100)	55 (100)	<0.0001**
Class I NYHA, N (%)	91 (46.19)	3 (5.45)	<0.0001**
Class II NYHA, N (%)	101 (51.26)	44 (80.00)	<0.0001**
Class III NYHA, N (%)	5 (2.53)	8 (14.54)	0.405**
HBP, N (%)	179 (90.86)	40 (72.72)	<0.001**
Stage I, N (%)	34 (17.25)	4 (7.27)	<0.0001**
Stage II, N (%)	114 (57.86)	35 (63.63)	<0.0001**
Stage III, N (%)	31 (15.73)	1 (1.81)	<0.0001**
Diabetes mellitus, N (%)	113 (57.36)	34 (61.81)	0.553**
Dyslipidemia, N (%)	172 (87.31)	41 (74.54)	0.021**
Peripheral venous disease, N (%)	45 (22.84)	9 (16.36)	0.300**
Dilated cardiomyopathy, N (%)	19 (9.64)	46 (83.63)	<0.001**
Chronic kidney disease, N (%)	32 (16.24)	19 (34.54)	0.008**
Lung disease, N (%)	22 (11.16)	13 (23.63)	0.018**
LVEF, N (%)	197 (100)	55 (100)	<0.001**
LVEF < 40%, N (%)	7 (3.55)	38 (69.09)	<0.0001**
LVEF ≤ 40%-50%, N (%)	21 (10.66)	14 (25.45)	0.236
LVEF ≥ 50%, N (%)	169 (85.78)	3 (5.45)	<0.0001**

The analysis of HF stages showed a higher frequency of HF stages I and II (46.193% and 51.269%) in Group HF-CT, while in Group HF-S/V, most patients were in stages II (80%) and III (14.54%). HBP stage III was more frequent in Group HF-CT (15.73% versus 1.81% in Group HF-S/V), while HBP stage II was more frequent in Group HF-S/V. Approximately 95% of patients in Group HF-S/V were found to have a reduced left ventricular ejection fraction (LVEF). Among them, 67.27% recorded values below 40% and 27.27% and between 40% and 50%. In Group HF-CT, less than 15% of the patients were in this situation.

Comorbidity index

**Figure 3:**
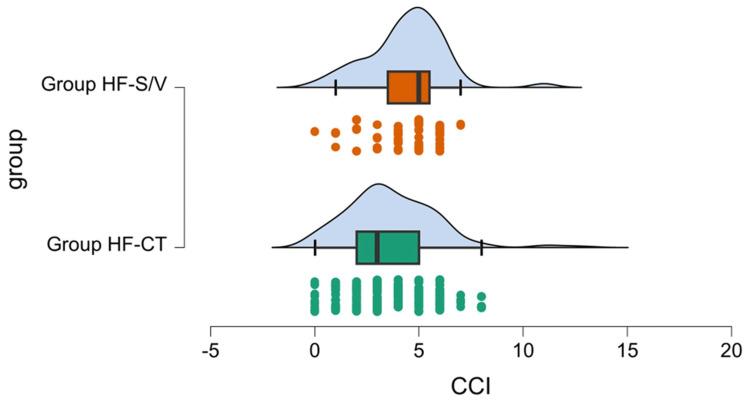
Average values of Charlson Comorbidity Index (CCI) scores, general distribution, individual trend, median, quartiles, and outliers. Group HF-S/V, patients undergoing treatment with sacubitril/valsartan; Group HF-CT, patients receiving conventional therapy

The mean value of the CCI score differed significantly between the two groups, with better results for Group HF-CT (3.685 ± 2.167 versus 4.400 ± 1.852, p = 0.006). For both groups, the means were in the range of 3-5. We used the raincloud plot (Figure 3) to present a more intuitive picture of the data (including the general distribution, individual trend, median, quartiles, and outliers).

The analysis of the score, by severity categories, showed a higher frequency of ≥5 score in Group HF-S/V (54.55% versus 31.47%), showing that in this group, the number of comorbidities and their severity is higher (Table 2). The contribution of age in increasing the CCI score does not determine the differences between the two groups (p = 0.103).

**Table 2:** Contingency tables of the two groups, depending on the number of comorbidities. Charlson category 1, CCI score of 1-2; Charlson category 2, CCI score of 3-4; Charlson category 3, CCI score of ≥5 CCI: Charlson Comorbidity Index

CCI categories	Group HF-CT	Group HF-S/V
Category 1, N (%)	58 (29.44)	9 (16.36)
Category 2, N (%)	77 (39.09)	16 (29.09)
Category 3, N (%)	62 (31.47)	30 (54.55)

Quality of life assessment

**Figure 4:**
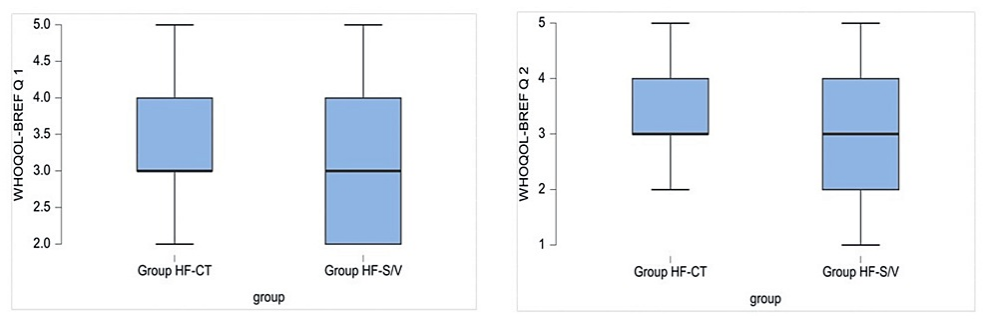
Graphic representation of the average values of the answers for questions Q1 and Q2 of the World Health Organization Quality of Life Brief Version (WHOQOL-BREF) questionnaire. Group HF-S/V, patients undergoing treatment with sacubitril/valsartan; Group HF-CT, patients receiving conventional therapy

Data analysis showed significant differences between groups on items Q1, "individual's general perception of quality of life," and Q2, "individual's general perception of his/her health," with better results in Group HF-CT (3.249 ± 0.817 versus 2.944 ± 0.834, p < 0.001, and 3.284 ± 0796 versus 2.981 ± 0.879, p < 0.001, respectively) (Figure 4).

It was found that less than 2% reported very good quality of life and very good health in both groups (Table 3). In Group HF-CT, 44.16% consider having a good quality of life, and 45.18% rate a state of health as good. In Group HF-S/V, 34.54% and 38.18%, respectively, consider the quality of life as poor and neither poor nor good. Regarding self-rated health, 30.90% rate their health as poor, and more than 1/3 rate their health as neither poor nor good. Only one patient in Group HF-S/V answered very good to both questions.

**Table 3:** Contingency tables regarding the answers to questions Q1 and Q2 (World Health Organization Quality of Life Brief Version {WHOQOL-BREF} questionnaire). Group HF-S/V, patients undergoing treatment with sacubitril/valsartan; Group HF-CT, patients receiving conventional therapy

Answer	Group HF-CT	Group HF-S/V	P-value	Group HF-CT	Group HF-S/V	P-value
	Q1	Q1		Q2	Q2	
Very poor, N (%)	-	-	-	-	1 (1.81)	-
Poor, N (%)	44 (22.34)	19 (34.54)	0.0016	39 (19.80)	17 (30.90)	0.003
Neither poor nor good, N (%)	63 (31.98)	21 (38.18)	<0.0001	66 (33.50)	20 (36.36)	<0.0001
Good, N (%)	87 (44.16)	14 (25.45)	<0.0001	89 (45.18)	16 (29.09)	<0.0001
Very good, N (%)	3 (1.52)	1 (1.81)	0.31	3 (1.52)	1 (1.81)	0.31

Quality of social relationships

**Figure 5:**
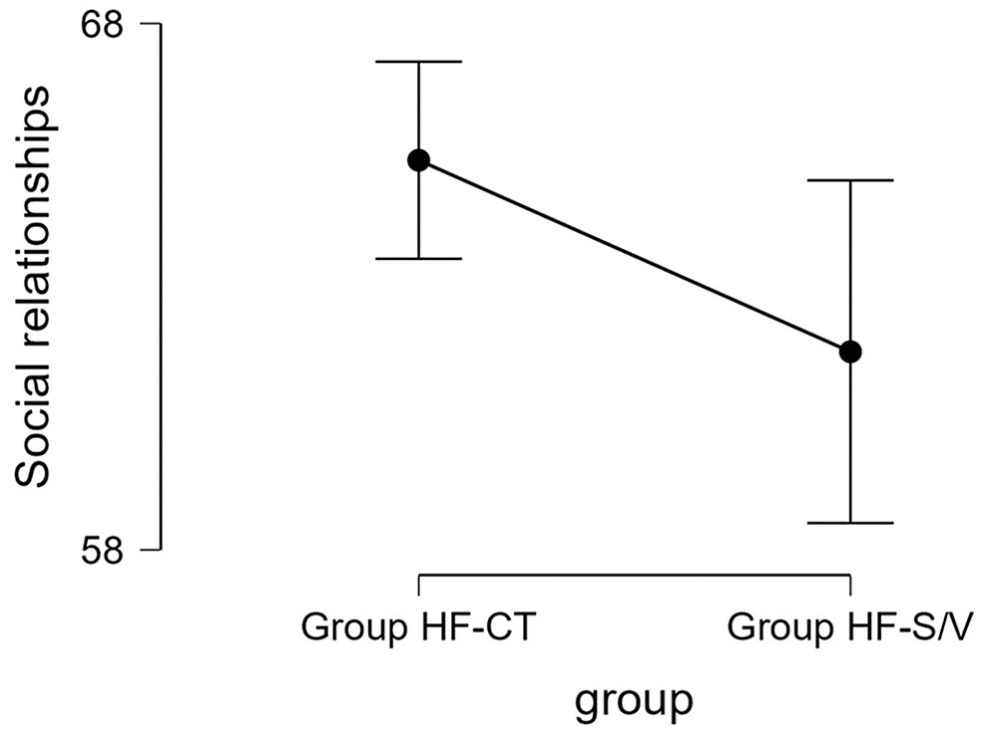
Mean values obtained for social relationships for the two groups. Group HF-S/V, patients undergoing treatment with sacubitril/valsartan; Group HF-CT, patients receiving conventional therapy

The mean values obtained per domain of social relationships were significantly better for Group HF-CT (65.762 ± 12.519 versus 61.266 ± 12.428, p = 0.024) (Figure 5).

The results shown in Table 4 indicate large differences between the two groups.

**Table 4:** Contingency tables regarding responses in social relationships. Group HF-S/V, patients undergoing treatment with sacubitril/valsartan; Group HF-CT, patients receiving conventional therapy

Social relationships	Group HF-CT	Group HF-S/V	P-value
Very poor, N (%)	1 (0.51)	0 (0.00)	-
Poor, N (%)	9 (4.57)	3 (5.46)	0.083
Moderate, N (%)	55 (27.92)	26 (47.27)	0.0013
Good, N (%)	111 (56.34)	25 (45.45)	<0.0001
Very good, N (%)	21 (10.66)	1 (1.82)	<0.0001

Using multivariate regression, we examined which factors influence these differences between the two groups. Table 5 shows the summary statistics of the regression model for the quality of social relationships. The regression model explains 24.5% of the total variability in social relationship quality and is statistically significant (F {12239} = 7.793, p < 0.001). Next, we inspected the model coefficients to determine which predictors contribute significantly to the explained variability.

**Table 5:** Statistics of the regression model for the quality of social relationships. F, the ratio of explained variation to unexplained variation; df, degrees of freedom

Model		Sum of squares	df	Mean square	F	P-value
H₁	Regression	12146.466	12	1012.206	7.793	<0.001
	Residual	31041.641	239	129.881		
	Total	43188.107	251			

For the quality of social relationships, only age and category of ≥5 of the Charlson Comorbidity Index contribute significantly as predictors. The treatment group (Group HF-CT versus Group HF-S/V) is not significantly associated with this quality of life dimension. For age, we found a significant and negative correlation with the quality of social relationships. Specifically, when keeping all the other predictors fixed (i.e., statistically controlling for the other predictors), the score on the quality of social relationship scale decreases by 0.294 points on average, for every one-year increase in age (or by approximately three points on average for every 10-year increase in age).

The effect of the Charlson category on the quality of social relationships of patients with HF is given by the difference between categories 1 and 3. Specifically, the patients with a category 3 on the Charlson Comorbidity Index show a decrease of 9.21 points in average on the quality of social relationships, when compared to patients with a category 1 of the Charlson Comorbidity Index (Table 6). This is a large effect (Cohen's d = 0.808).

**Table 6:** Statistically significant predictors of social relationships. Charlson category 2, CCI score of 3-4; Charlson category 3, CCI score of ≥5; Group HF-S/V, patients undergoing treatment with sacubitril/valsartan HBP, high blood pressure; LVEF, left ventricle ejection fraction; CCI, Charlson Comorbidity Index

Model		Unstandardized	Standard error	t	P-value
H₁	Group HF-S/V	0.217	2.765	0.079	0.937
	Age	-0.294	0.087	-3.372	<0.001
	Sex (male)	1.292	1.526	0.847	0.398
	Living area (city area)	2.124	1.590	1.336	0.183
	HPB (I)	1.255	2.824	0.445	0.657
	HPB (II)	-0.404	2.272	-0.178	0.859
	HPB (III)	-2.263	3.024	-0.748	0.455
	Dyslipidemia (No)	-2.193	2.041	-1.074	0.284
	LVEF (≤40%-50%)	-0.805	2.913	-0.276	0.783
	LVEF (≥50%)	1.018	3.142	0.324	0.746
	Charlson category 2	-3.354	2.171	-1.545	0.124
	Charlson category 3	-9.209	2.567	-3.587	

**Figure 6:**
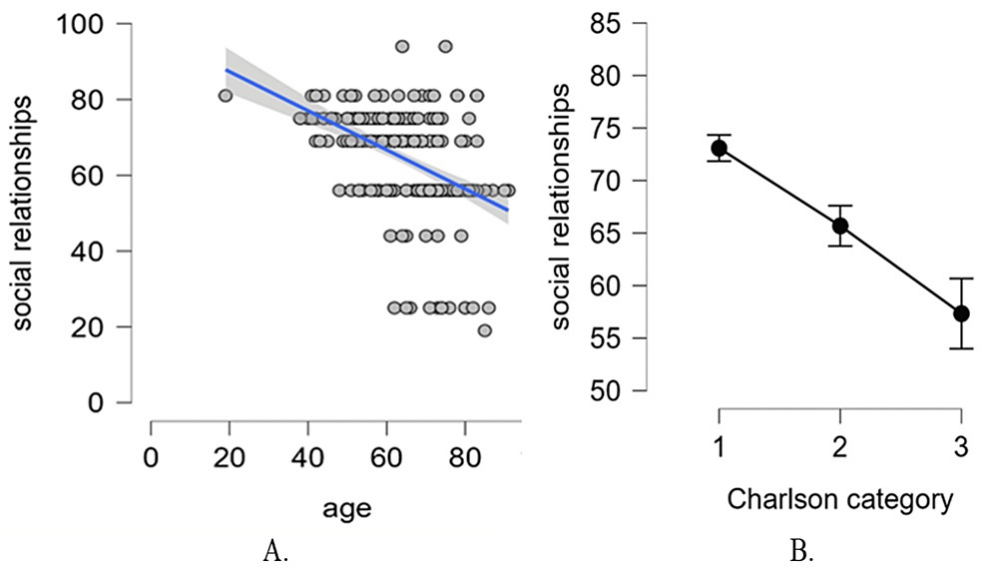
(A) Scatter plot of age and the quality of social relationships, with regression line. (B) The effect of the Charlson category on the quality of social relationships (error bars with confidence intervals).

The significant effects are depicted in Figure 6.

Environmental well-being

**Figure 7:**
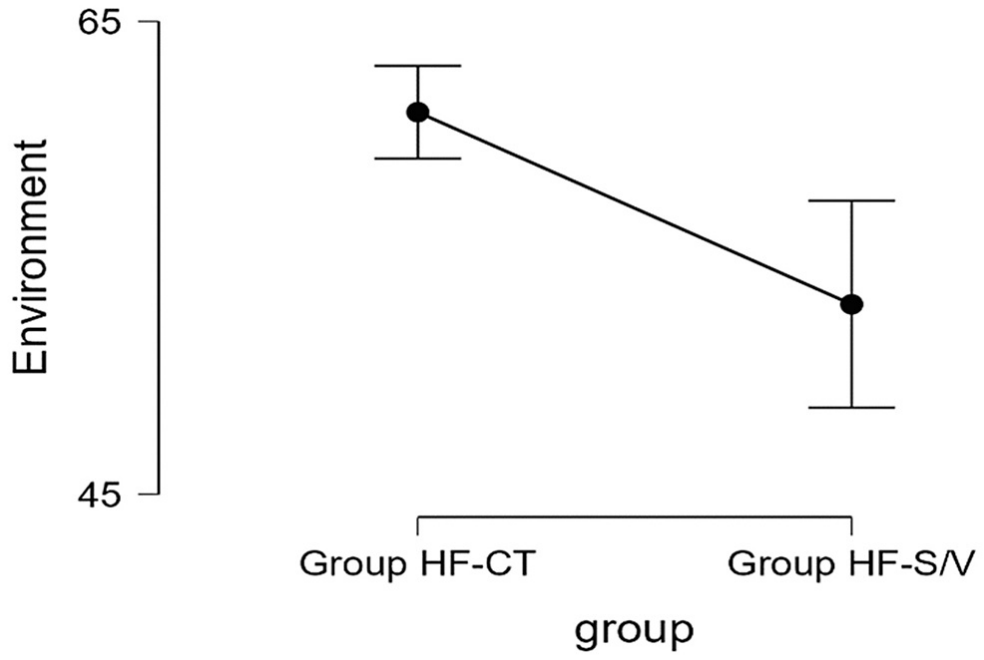
Average values obtained for the environmental well-being domain for the two groups. Group HF-S/V, patients undergoing treatment with sacubitril/valsartan; Group HF-CT, patients receiving conventional therapy

The mean values obtained in relation to the environment (Figure 7) were significantly better for Group HF-CT (61.333 ± 13.461 versus 51.719 ± 16.769, p < 0.001).

The results on the relationship with the environment also showed large differences between groups, especially in moderate and good responses (Table 7).

**Table 7:** Contingency tables on patient responses in relation to the environment. P-value, chi-square method; Group HF-S/V, patients undergoing treatment with sacubitril/valsartan; Group HF-CT, patients receiving conventional therapy

Environmental relation	Group HF-CT	Group HF-S/V	P-value
Poor, N (%)	29 (14.72)	17 (30.90)	0.07
Moderate, N (%)	34 (17.26)	16 (29.09)	0.01
Good, N (%)	133 (67.51)	22 (40.00)	<0.0001
Very good, N (%)	1 (0.51)	0 (0.00)	-

The regression model explains 43.1% of the total variability in environmental well-being, and it is statistically significant (F {12239} = 16.821, p < 0.001), as shown in the table above. Further, we inspected the model coefficients to see which of the predictors contributed significantly to the explained variability (Table 8).

**Table 8:** Statistics of the regression model for the quality of the relationship with the environment. F, ratio of explained variation to unexplained variation; df, degrees of freedom

Model		Sum of squares	df	Mean square	F	P-value
H₁	Regression	25199.071	12	2099.923	16.821	<0.001
	Residual	29836.591	239	124.839		
	Total	55035.663	251			

Similar to the quality of social relationships, age and category 3 are the only significant predictors of environmental well-being. The treatment group (Group HF-CT versus Group HF-S/V) is not significantly associated with this quality of life dimension. For age, a significant negative correlation with environmental well-being was found. Specifically, when keeping all the other predictors fixed (i.e., statistically controlling for the other predictors), the score on the environmental well-being scale decreases by 0.33 points on average, for every one-year increase in age (or by approximately 3.3 points on average for every 10-year increase in age).

The effect of the Charlson category on the environmental well-being of patients with HF is given by the difference between categories 1 and 3 (Table 9). Specifically, the patients with a category 3 on the Charlson Comorbidity Index show a decrease of 12.3 points on average on environmental well-being, when compared to patients with a category 1 of the Charlson Comorbidity Index. This is a very significant effect (Cohen's d = 1.101).

**Table 9:** Statistically significant predictors of environmental well-being. Charlson category 2, CCI score of 3-4; Charlson category 3, CCI score of ≥5; Group HF-S/V, patients undergoing treatment with sacubitril/valsartan HBP, high blood pressure; LVEF, left ventricle ejection fraction; CCI, Charlson Comorbidity Index

Model		Unstandardized	Standard error	t	P-value
H₁	Group HF-S/V	-0.452	2.711	-0.167	0.868
	Age	-0.353	0.085	-4.134	<0.001
	Sex (Male)	0.376	1.496	0.251	0.802
	Living area (city area)	2.473	1.559	1.587	0.114
	HBP (I)	-0.150	2.769	-0.054	0.957
	HBP (II)	-2.061	2.227	-0.926	0.356
	HBP (III)	-1.324	2.965	-0.447	0.656
	Dyslipidemia (No)	-1.625	2.001	-0.812	0.418
	LVEF (≤40%-50%)	-1.671	2.856	-0.585	0.559
	LVEF (≥50%)	3.609	3.081	1.172	0.243
	Charlson category 2	1.912	2.129	0.898	0.370
	Charlson category 3	-12.296	2.517	-4.885	<0.001

**Figure 8:**
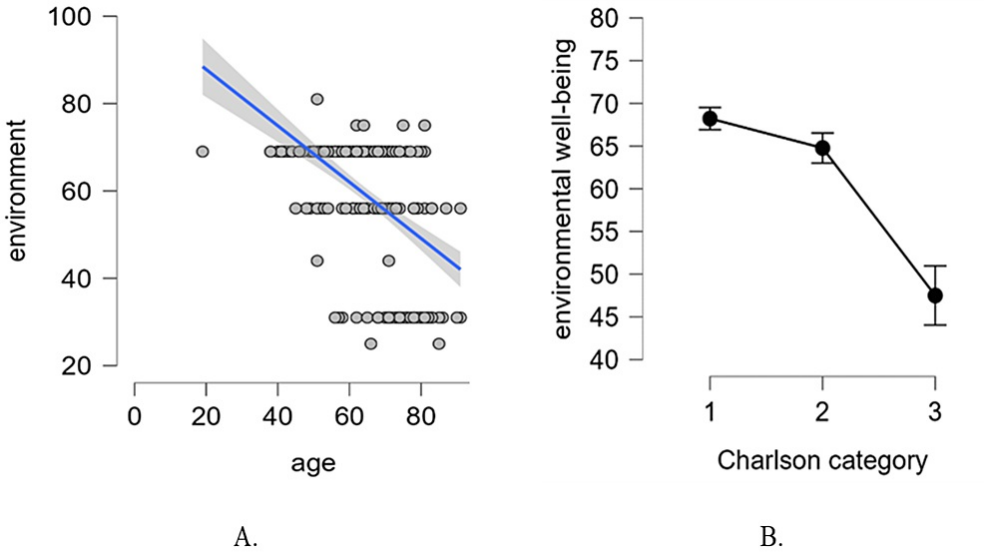
(A) Scatter plot of age and environmental well-being, with regression line. (B) The effect of the Charlson category on environmental well-being (error bars with confidence intervals). Charlson category 1, CCI score of 1-2; Charlson category 2, CCI score of 3-4; Charlson category 3, CCI score of ≥5 CCI: Charlson Comorbidity Index

The significant effects are depicted in Figure 8.

## Discussion

Quality of life assessment tools are essential in assessing health status [16]. HF affects all dimensions of patients' quality of life (physical, emotional, social, and environmental). Previous studies assessing the quality of life of patients with HF compared to controls (patients without HF) have shown that age, gender [17,18], educational status, and occupation are the main sociodemographic factors with a major impact on quality of life. Clinical factors such as NYHA functional class, comorbidities, and paraclinical factors (LVEF) have been identified as predictive factors in some studies [19].

The aspects assessed, by applying the WHOQOL-BREF questionnaire in this study, are social relationships (relationships with and support from friends and sex life) and environmental well-being (daily safety, financial needs, patient satisfaction with own living conditions, and access to transport and health services), as well as the identification of predictors of this dimension. Psychosocial stress and environmental health have effects on cardiovascular risk factors (HBP, diabetes, and obesity) [6].

Recommendations published in 2022 [20] indicate the use of S/V in patients with heart failure with reduced ejection fraction (HFrEF) and HF class II/III symptoms to improve the prognosis of these patients. The effects of S/V administration (approved for the relief of HF symptoms) have been shown to improve plasma natriuretic peptide levels and myocardial remodeling [5]. The Food and Drug Administration (FDA) has approved S/V administration in treating patients with heart failure with preserved ejection fraction (HFpEF). The Prospective Comparison of ARNI with ARB Global Outcomes in HF With Preserved Ejection Fraction (PARAGON-HF) study also mentioned beneficial effects on quality of life and renal function [7].

The results of this study showed no significant differences between the two groups regarding age, gender, and ponderal status (p > 0.05). Approximately 95% of patients in Group HF-S/V have HF stages II and III (versus 53.81%). Of patients with HFrEF (LVEF ≤ 40), 67.27% are in Group HF-S/V, compared to Group HF-CT, where 85.78% are normal LVEF. Dyslipidemia has increased incidence in both study groups. Chronic kidney disease and lung diseases are found in approximately double the percentage in Group HF-S/V. Dilated cardiomyopathy is present in 83.64% of patients receiving angiotensin receptor-neprilysin inhibitor (ARNI) treatment (S/V is the only drug known in this class). A study published by Băjenaru et al. (2022, N = 60) [21] assessed the quality of life in geriatric patients (without any acute medical condition, major neurocognitive disorder, angina pectoris, uncontrolled HBP, and heart arrhythmias) using the same questionnaire (with a mean age of 71.95 ± 5.98) and obtained a mean of 72.64 ± 11.60 for the social relation domain. In this study, the values obtained for the same domain were 65.762 ± 12.519 (Group HF-CT) and 61.266 ± 12.428 (Group HF-S/V). Also, in the study of geriatric patients, the mean score for the relationship with the environment was 78.59 ± 10.84. In our study, the presence of HF determines a greater decrease in the score in social relationships (Group HF-CT, 61.333 ± 13.461; Group HF-S/V, 51.719 ± 16.769). In the case of Group HF-S/V, the score reaches approximately 50% of the maximum value, and 65.8% of the score was obtained for geriatric patients. As can be seen, the presence of HF leads to a decrease in the score for social relationships, even in the context of a much younger age, in the case of the group with S/V treatment of approximately 10 years. Explanations may stem from the increased incidence in this NYHA stage II HF group of dilated cardiomyopathy in 83.64% and the presence of LVEF of ≤40%. The CCI is significantly higher in the S/V group. All these cause a reduction in physical performance and, therefore, affect social and environmental relationships.

The multiple regression analysis performed in our study highlights age and the CCI as predictors of quality of life for the social relationships domain. For each 10-year increase in age, the score decreases by three points. The effect of the CCI on the quality of social relationships in HF patients is given by the difference between categories 1 and 3. HF patients' category 3 quality of life score (CCI score of ≥5) in this domain was 9.21 points lower on average than those in category 1 (CCI score of 1-2).

The same factors have been shown to influence the relationship with the environment. Thus, for every 10-year increase in age, the score decreases by 3.3 points; the CCI score of ≥5 causes the score on the relationship with the environment domain to decrease by 12.3 points.

The treatment group (Group HF-CT versus Group HF-S/V) is not significantly associated with this dimension of the quality of life. The responses on the individual's overall perception of the quality of life and the individual's overall perception of his/her health are significantly different depending on the study group.

In our study, gender, residence, HBP stages, and LVEF do not correlate with monitored quality of life domains.

Ewnetu Tarekegn et al. published the results of a study (2021) in which they assessed the quality of life of HF patients. The results showed a low mean score in environmental health and general quality of life domains. In the domain of social relationships, the score was moderately low. Similar to our study, in Ewnetu Tarekegn et al.'s study, age negatively affects social and environmental relationships [22].

The study published in 2023 by Lawson et al. followed patients' quality of life (N = 23553 patients) over one year. It supports the importance of the routine use of health questionnaires to provide data to enable patient-centered treatment; the results also show that quality of life scores are independent of age, sex, and ejection fraction [23]. Similarly, the study published in 2023 by Jarab et al. (N = 427) supports the decrease in the quality of life of HF patients; the variables that condition this aspect are patients' income, associated comorbidities, and HF stage [24].

The multiple linear regression analysis performed on a cohort of 383 patients (Mulugeta et al., 2023) [25] identifies factors such as age, comorbidities (diabetes), and social support scores that influence the quality of life. Another study was conducted by Comín-Colet et al. in 2016, comparing HF patients with the general population (n = 1037). Multivariable linear regression analysis identified age, female sex, HF stages, and increased comorbidity index as factors that influenced the quality of life of HF patients [26]. Lastly, Costa et al. conducted a study in 2020 on 142 patients hospitalized with HF in Bangladesh. The study identified several factors that correlated with HF, including gender, marital status, education level, income, smoking, residence, and body mass index (BMI) [27].

In order to get an overview of the quality of life of HF patients, Moradi et al. (2020) [28] conducted and published a meta-analysis (period analyzed from January 2000 to December 2018, 70 studies), which aimed to determine the quality of life score using the 36-Item Short-Form Health Survey (SF-36) questionnaire, which, in addition to the overall value, provides information on different dimensions of life (physical functioning, physical role, bodily pain, general health, vitality, social functioning, emotional role, and mental health) [29]. The survey results show a marked decrease in the average score (44.1), with differences between geographical areas [28]. In our study, the results indicate a decrease in the quality of life of patients with HF, on the dimensions assessed, with the results associated with Group HF-S/V being lower than in Group HF-CT. The results of our study indicate a decrease in the quality of life of HF patients (regardless of treatment) on the dimensions assessed, but these do not correlate with the residential environment. Aspects are different due to the fact that in the meta-analysis, we refer to different geographical areas, not rural/urban areas.

Strengths and limitations of the study

This study is among the few studies that evaluate social relationships and the relationship with the environment of HF patients, aspects that are frequently neglected, and includes a significant number of patients. The main limitations of the study are primarily due to the cross-sectional design, without follow-up or optimized treatment information, and the subjective nature of the self-administered quality of life questionnaire used. Another limitation of the study is the relatively small number of patients in Group HF-S/V (the number explained by the need to respect the protocol of treatment initiation according to the left ventricular ejection fraction).

## Conclusions

Social relationships and the relationship with the environment are affected in HF patients and correlate with age and comorbidity index, regardless of the type of therapy. Future studies with a follow-up and multicenter design including a large number of patients are needed to confirm these results. Quality of life assessment questionnaires identify weaknesses highlighted by patients; they can be used as a starting point to improve the quality of care and patient-centered health policies.
